# Psychometric properties of the 25-item National Eye Institute Visual Function Questionnaire (NEI VFQ-25), Japanese version

**DOI:** 10.1186/1477-7525-3-65

**Published:** 2005-10-26

**Authors:** Yoshimi Suzukamo, Tetsuro Oshika, Mitsuko Yuzawa, Yoshihiro Tokuda, Atsuo Tomidokoro, Kotaro Oki, Carol M Mangione, Joseph Green, Shunichi Fukuhara

**Affiliations:** 1 Department of Epidemiology and Healthcare Research, Kyoto University, Yoshida -Konoe-cho, Sakyo-ku, Kyoto, Japan; 2 Institute for Health Outcomes and Process Evaluation Research, Kudan Building 2nd floor, Iidabashi 1-4-7, Chiyoda-ku, Tokyo, Japan; 3 Department of Ophthalmology, Institute of Clinical Medicine, University of Tsukuba, 1-1-1 Tennoudai, Tsukuba, Ibaraki, Japan; 4 Department of Ophthalmology, Nihon University Hospital, 1-8-13, Kanda-Surugadai, Chiyoda-ku, Tokyo, Japan; 5 Inouye Eye Hospital, 4-3, Kanda-Surugadai, Chiyoda-ku, Tokyo, Japan; 6 Department of Ophthalmology, Graduate School of Medicine, the University of Tokyo, 7-3-1, Hongo, Bunkyo-ku, Tokyo, Japan; 7 Oki Eye Surgery Center, Sojukai Medical foundation, 2-17-1, Ikebukuro, Toshima-ku, Tokyo, Japan; 8 Division of General Internal Medicine and Health Services Research, Department of Medicine, David Geffen School of Medicine at UCLA, 911 Broxton Plaza, Room 313, Los Angeles, CA, USA; 9 Graduate School of Medicine, University of Tokyo, 7-3-1, Hongo, Bunkyo-ku, Tokyo, Japan

## Abstract

**Background:**

The importance of evaluating the outcomes of health care from the standpoint of the patient is now widely recognized. The purpose of this study is to develop and test a Japanese version of the National Eye Institute Visual Function Questionnaire (NEI VFQ-25).

**Methods:**

A Japanese version was developed with a previously standardized method. The questionnaire and optional items were completed by 245 patients with cataracts, glaucoma, or age-related macular degeneration, by 110 others before and after cataract surgery, and by a reference group (n = 31). We computed rates of missing data, measured reproducibility and internal consistency reliability, and tested for convergent and discriminant validity, concurrent validity, known-groups validity, factor structure, and responsiveness to change.

**Results:**

Based on information from the participants, some items were changed to 2-step items (asking if an activity was done, and if it was done, then asking how difficult it was). The near-vision and distance-vision subscales each had 1 item that was endorsed by very few participants, so these items were replaced with items that were optional in the English version. For example, more than 60% of participants did not drive, so the driving question was excluded. Reliability and validity were adequate for all subscales except driving, ocular pain, color vision, and peripheral vision. With cataract surgery, most scores improved by at least 20 points.

**Conclusion:**

With minor modifications from the English version, the Japanese NEI VFQ-25 can give reliable, valid, responsive data on vision-related quality of life, for group-level comparisons or for tracking therapeutic outcomes.

## Background

The importance of evaluating the outcomes of health care from the standpoint of the patient is now widely recognized. Measures of health-related quality of life (HRQOL) have been used to track outcomes for many eye diseases [1-6]. HRQOL refers to health status in the physical, mental, and social domains, and to the effect of a disease, its symptoms, and treatments on patients' lives. Conventional clinical measures such as visual acuity and visual field assessments do not fully capture the influence of visual disability on daily visual functioning and on abilities to perform activities of daily living that are valued by patients.

In response to a need for a vision-targeted measure of quality of life, the National Eye Institute (NEI) funded the development of such an instrument in the mid-1990s. The resulting 51-item questionnaire is known as the National Eye Institute Visual Function Questionnaire (NEI VFQ) [7,8]. To lessen the burden on respondents and to improve data quality, a shorter version was developed: the NEI VFQ-25 [9]. The NEI VFQ-25 has 25 items that measure vision-targeted HRQOL and are grouped into 12 subscales: general health (GH, 1 item); general vision (GV, 1 item); ocular pain (OP, 2 items); difficulty with near-vision activities (NV, 3 items); difficulty with distance-vision activities (DV 3 items); limitation of social functioning due to vision (SF, 2 items); mental health problems due to vision (MH, 4 items), role limitations due to vision (RL, 2 items); dependency on others due to vision (DP, 3 items); driving difficulties (DR, 2 items); difficulty with color vision (CV, 1 item); and difficulty with peripheral vision (PV, 1 item). Each subscale score is converted to a score between 0 and 100, and higher scores indicate better vision-specific HRQOL. The composite VFQ-25 score is the mean score of all items except for the general health item. The VFQ-25 has adequate reliability and validity, and subscale scores from the shorter form correlate highly with scores on the original long version. This questionnaire has been translated into Italian, French, Spanish, and German, and validated [10-13], and it has been widely used to describe the HRQOL of patients with ocular disease and to assess the treatment of ocular disease [14-20].

We developed a Japanese version of the NEI VFQ-25 (Appendix [see additional file 1]). and evaluated its psychometric characteristics. We investigated three points in particular. First, we looked at each question item in the Japanese version quantitatively and qualitatively, taking into consideration Japanese lifestyles, and made the necessary adaptations. Second, although composite NEI VFQ-25 scores can be computed, there is no published evidence of this scale's uni-dimensionality. Therefore, on the basis of the Japanese version's factor structure and other psychometric characteristics, we propose a particular combination of subscales that can be used to compute an appropriate composite score. Third, research on the responsiveness of the NEI VFQ-25 is limited [4,21], so we quantified its responsiveness, using data obtained before and after cataract surgery.

## Methods

### Development of the Japanese version

One of us (CMM) was a developer of the original NEI VFQ-25. The Japanese version was developed in conformance with standard methods that have been adopted internationally [22], including forward translation, back-translation, examination of the translation quality and adjudication by bilingual speakers, and a pilot test on 15 persons. One item was changed to make then more appropriate to Japanese life style and culture (details below). The content of the translated questionnaire was reviewed by one of the original developers of the English version, and the Japanese version was considered appropriate for administration in a psychometric field test.

### Study design and population

Two groups of patients were studied. The first group was a convenience sample of 276 outpatients who visited the departments of ophthalmology at 5 hospitals. To participate, patients had to be 21 years of age or older, had to have clinical evidence of age-related cataracts, glaucoma, or age-related macular degeneration (ARMD), and had to have been seen at least twice in the past 3 months at the participating hospital. For patients with cataracts, the inclusion criteria were having cataracts in both eyes and 20/30 or worse visual acuity in the better eye. Inclusion criteria for patients with glaucoma were binocular primary open-angle glaucoma, binocular abnormalities as measured with a Humphrey field analyzer, defects in the optic nerve, at least one documented instance (in each eye) of intraocular pressure greater than 21 mmHg, and no incisional surgery for treatment of glaucoma during the previous 3 months. For patients with ARMD, there were three inclusion criteria: having at least one of the following 5 conditions: abnormal retinal pigmented epithelium, sub-retinal neovascular membrane, disciform scar, previous laser treatment to the macula, or geographic atrophy involving the fovea; having small drusen in other areas; and binocular involvement. Also included in Sample 1 was a reference group of patients with refractive error only and hospital employees.

The second sample consisted of 110 patients who had been recruited from 6 different departments of ophthalmology and were scheduled for bilateral cataract surgery (phacoemulsification and implantation of foldable intraocular lenses). Inclusion criteria for these patients were bilateral cataracts and preoperative corrected visual acuity of 20/30 or better in both eyes.

Attending physicians explained the research and ethical considerations to the participants, who then indicated their understanding by signing an informed-consent form. This study was done in accord with the Declaration of Helsinki.

### Data collection

All surveys were administered by a trained interviewer. The interviewers had no direct involvement in the medical care of the patients. The interviews included the Japanese version of the NEI VFQ-25 and 14 optional items about aspects of vision-specific HRQOL (which were not presented to patients who underwent cataract surgery), and SF-36 to measure general HRQOL [23,24].

The attending physician recorded, on a structured form, the type of eye disease, duration of disease, uncorrected vision, maximally refracted vision, vision with habitual correction, and ocular pressure. In addition, severity of age-related cataracts was graded with the Lens Opacities Classification System (LOCS) III (slit lamp, standard testing conditions [25]), and in participants with glaucoma visual field was assessed with a Humphrey field analyzer 30-2. In patients with ARMD, the type of ARMD and the size and location of absolute scotoma were recorded. The data were managed by ID number, and were analyzed in a way that maintained the participants' privacy.

### Statistical analysis

All statistical analyses were done with SPSS version 12 for Windows (SPSS Inc, Chicago, IL).

### Descriptive analysis and item analysis

The item analysis was done using the data from the multi-condition group (Sample 1). The percentage of missing values was examined for each item. We also examined whether each item's distribution of responses was strongly skewed (large ceiling effect or floor effect).

### Reliability

Cross-sectional data from the multi-condition group (Sample 1) were used to quantify reliability. Cronbach's alpha coefficient [26] was used as the index of internal consistency for each subscale. To quantify test-retest reliability, intraclass correlation coefficients [27] were used. The test-retest data were obtained from clinically stable patients with age-related cataracts, in surveys done 2 weeks apart.

### Validity

The use of multi-trait analysis to evaluate convergent and discriminant validity has been described previously in detail [28]. What follows is a brief summary of the method: Each item is hypothesized to belong to only one multi-item subscale. For each item, correlations between the score on that item and the scores on all the subscales are computed. Then, for each item, if the correlation between the score on that item and the score on the subscale to which that item belongs is 0.4 or higher, that item is said to have "passed" the test of convergent validity. Also for each item, if the correlation between the score on that item and the score on the subscale to which that item belongs is greater than the correlations between the score on that item and the scores on all the subscales to which it that item does not belong, then that item is said to have "passed" the test of discriminant validity [29].

To assess concurrent validity, we computed correlations between scores on the NEI VFQ-25 and on the SF-36 subscales. We hypothesized that the NEI VFQ-25 "mental health", "social functioning", "role difficulties" and "dependency" scores would be associated more strongly with the SF-36 subscale scores that measured similar domains.

The subscale scores of participants with poor visual acuity were compared to those of participants with better visual acuity. Also, by analysis of variance, the subscale scores were compared among those with age-related cataracts, ARMD, and the reference group. In addition, scores on the peripheral-vision subscale in the patients with glaucoma were compared to those in the reference group. We also computed the correlations between subscale scores and visual acuity with habitual correction in the better and worse eye and deficits in visual fields as measured by the Humphrey Field Analyzer 30-2 in the better and worse eye.

Finally, we used factor analysis to assess the uni-dimensionality of the scale, in preparation for computing a composite score. Factor analysis was done using 10 subscales ('General Health' and 'Driving' were not included), with the maximum-likelihood solution and promax rotation. The 'Driving' subscale was not included because more than 60% of the responses on this subscale were missing.

### Responsiveness

Responsiveness was studied using data from the reference group and from the patients who completed the survey before and 2 months after cataract surgery. Differences related to cataract surgery were analyzed with Student's t-test for paired data, and with the responsiveness statistic of Guyatt [30]. The responsiveness statistic is the ratio of the clinically important difference (sometimes denoted by the Greek letter delta in sample-size calculations) to the variability in stable subjects (the square root of twice the mean square error).

## Results

### Translation and pilot test

On the basis of the translations and discussions among the developers, one item was changed to conform better to Japanese norms. In The item "Because of your eyesight, how much difficulty do you have visiting with people in their homes, at parties, or in restaurants?", "visit at parties" was changed to "going to gatherings". Also, when a pilot test was done in 5 subjects without eye disease and 10 subjects with eye conditions, we found no expression equivalent to 'not applicable'. Therefore each item was rewritten so that it had a stem, in which the participants were asked whether they did the activity. If they indicated that they did the activity, then they were asked about the degree of difficulty in doing it. If they indicated that they did not do the activity, then they were asked whether this was due to vision problems. All such changes were discussed with, and approved by, one of the original NEI VFQ developers (CMM).

### Subjects

Sample 1 had 276 participants and Sample 2 had 110. All those in Sample 1 were included in the analytic sample. In Sample 2, 4 patients did not answer the questionnaire and 11 did not respond after cataract surgery, thus 95 patients were in the analytic sample for this group. The characteristics of the participants are shown in Table 1.

**Table 1:** Sociodemographic and clinical characteristics of the two samples

	Sample 1, n = 276	Sample 2, n = 95
Mean of age (range)	66.8 (21 to 95)	71.9 (52 to 86)
Female (%)	141 (51.0)	71 (74.7)
Visual acuity (Snellen fraction)		
Better eye, mean (range)	20/120 (20/13 to 20/2000)	20/110 (20/16 to 20/2000)
Worse eye, mean (range)	20/200 (20/13 to 20/2000)	20/200 (20/20 to 20/2000)
Chronic eye disease, number (%)		
Age-related cataract	96 (34.8)	95 (100)
Glaucoma	69 (25.0)	Not applicable
Age-related macular degeneration	80 (29.0)	Not applicable
Normal reference	31 (11.2)	Not applicable
Medical comorbidities*		
0	104 (37.7)	Not applicable
1	97 (35.1)	Not applicable
2 or more	75 (27.2)	Not applicable

Of the patients with ARMD, 7 had only dry change in both eye, 8 had exudative changes in one eye, 56 patients had exudative changes in both eyes, and the status 9 patients was unknown. In the patients with glaucoma, their mean dB threshold values were -12.8 for the right eye and -12.9 for the left eye with Humphrey 30-2 threshold perimetry test. In the cataract patients of sample 1, the mean values measured by LOCS III were 2.04 for nuclear color, 2.07 for nuclear opalescence, 2.42 for cortical opacity, and 1.84 for posterior subcapsular opacity in better eye. The mean values in sample-2 patients were 2.76, 2.78, 3.31 and 2.18 respectively.

### Item analysis

Percentages of missing values for each item and proportions of responses at the floor (the lowest possible score) and ceiling (the highest possible score) are shown in Table 2. 'Finding objects on crowded shelf' which was included in the 'Near Vision' subscale was not endorsed by 28% of the respondents, while 'going out to movies/plays' which was included in the 'Distance Vision' subscale was not endorsed by 32% of the sample. Three items each from the 'Near Vision' and 'Distance Vision' subscales in the optional item pool were included in the questionnaire (NV: reading small print, reading mail/bills, shaving/styling hair, DV: recognizing faces in room, participating in sports, seeing television). Subsequently, items with low rates of missing data were substituted for those with high rates, as long as the percentage of responses at the ceiling or floor did not exceed 50%. The result was that 'reading small print' was selected for the 'Near Vision' subscale and 'seeing television program' was selected for the 'Distance Vision' subscale.

**Table 2:** Results of item analysis. Number and percentage of missing data and of responses at the floor and ceiling (n = 276)

Subscale and Item	Missing Number (%)	Floor Number (%)	Ceiling Number (%)
General health: GH			
5-level health rating	1 (<1)	11 (4)	7 (3)
General vision: GV			
5-level general vision	2 (<1)	6 (2)	1 (<1)
Near vision: NV			
Reading normal newsprint	16 (6)	49 (18)	38 (14)
See well up close	36 (13)	43 (16)	43 (16)
Finding objects on crowded shelf	78 (28)	31 (11)	47 (17)
Distance vision: DV			
Going out to movies/plays	88 (32)	72 (26)	37 (13)
Going down stairs at night	24 (9)	38 (14)	36 (13)
Reading street signs	11 (4)	27 (10)	53 (19)
Driving: DR			
Daylight familiar places	169 (61)	43 (16)	39 (14)
Driving at night	221 (80)	15 (5)	7 (3)
Peripheral vision: PV			
Seeing objects off to side	6 (2)	8 (3)	47 (17)
Color vision: CV			
Difficulty matching clothes	27 (10)	5 (2)	158 (57)
Ocular pain: OP			
Amount pain	1 (<1)	2 (<1)	122 (44)
Amount time: pain	0	7 (3)	189 (69)
Role limitations: RL			
Accomplish less	1 (<1)	31 (11)	85 (31)
Limited in endurance	3 (1)	29 (11)	105 (38)
Dependency: DP			
Need much help from others	0	32 (12)	143 (52)
Stay home most of time	1 (<1)	38 (14)	127 (46)
Rely too much on other's word	0	29 (11)	154 (56)
Social function: SF			
Seeing how people react	40 (14)	22 (8)	64 (23)
Visiting others	25 (9)	22 (8)	98 (36)
Mental health: MH			
Amount true: frustrated	1 (<1)	30 (11)	117 (42)
Amount true: embarrassment	1 (<1)	31 (11)	134 (49)
Amount true: no control	2 (<1)	57 (21)	86 (31)
Amount true: worry	1 (<1)	40 (15)	32 (12)
Optional items			
Near vision: NV			
Reading small print	6 (2)	53 (19)	36 (13)
Reading mail/bills accurately	34 (12)	41 (15)	48 (17)
Shaving/styling hair/makeup	3 (1)	3 (1)	150 (54)
Distance vision: DV			
Recognizing faces in room	19 (7)	25 (9)	78 (28)
Participating in sports/outdoors	105 (38)	41 (15)	62 (23)
Seeing television program	5 (2)	17 (6)	91 (33)

More than 60% of the answers were missing for the 'Driving' subscale, which was much higher than the 16% and 31% obtained from surveys done in the United States.

### Reliability

Cronbach's alpha (the index of internal consistency reliability) was 0.7 or higher for almost all of the subscales. It was lower for the 'Ocular Pain' and 'Driving' subscales. With regard to test-retest reliability, the intraclass correlation coefficient was 0.7 or higher for all of the subscales except 'General Health', 'General Vision', and 'Peripheral Vision' (Table 3). These values are considered to indicate adequate reliability for group-level comparisons [31]. Substitution of items in the 'Near Vision' and 'Distance Vision' subscales (described above) did not affect the reliability of those subscales.

**Table 3:** Internal consistency and test-retest reliability of NEI VFQ-25 subscales

	Number of items	Cronbach's alpha	Intraclass correlation, for test-retest reliability	Range of item-scale correlations	Convergent validity*	Discriminant validity**
General health	1	NA***	0.51	NA	NA	NA
General vision	1	NA	0.48	NA	NA	NA
Near vision****	3	0.87 (0.85)	0.76 (0.69)	0.65 – 0.75 (0.69 – 0.73)	100 (100)	93.9 (100)
Distance vision****	3	0.84 (0.79)	0.85 (0.69)	0.67 – 0.75 (0.57 – 0.69)	100 (100)	90.9 (100)
Driving	2	0.58	0.99	0.49 – 0.49	100	50.0
Peripheral vision	1	NA	0.62	NA	NA	NA
Color vision	1	NA	0.74	NA	NA	NA
Ocular pain	2	0.44	0.75	0.28 – 0.28	0	81.8
Vision-specific						
Role limitation	2	0.82	0.88	0.70 – 0.70	100	90.9
Dependency	3	0.87	0.90	0.71 – 0.82	100	97.0
Social function	2	0.74	0.88	0.58 – 0.58	100	59.1
Mental health	4	0.84	0.94	0.62 – 0.75	100	90.9
25-item composite	25	0.96	0.94	NA	NA	NA

* The percentage of items that passed the test of convergent validity (as described in the text).

** The percentage of items that passed the test of discriminant validity (as described in the text).

*** NA: Not applicable to single-item scales

**** Scores were recomputed after the substitution of items described in the text. Results of the recomputations are in parentheses.

### Validity

All items passed the test of convergent validity, and 80% passed the test of discriminant validity. The success rates for the 'Near Vision' and 'Distance Vision' subscales were higher after item substitution than before (Table 3).

For concurrent validity, there were high correlations between scores on the NEI VFQ-25 subscales and similar domains of the SF-36 (Table 4). For example, The highest correlations were with the "Vitality" and "Mental Health" subscales, followed by the "Role Physical" and "Role Emotional" subscales. Correlations with the "Bodily Pain" and "Physical Functioning" subscales were low.

**Table 4:** Correlation of NEI-VFQ 25 subscales and the SF-36

	SF-36							
	
	Physical Functioning	Role Physical	Bodily Pain	General Health	Vitality	Social Functioning	Role Emotional	Mental Health
General health	.308	.220	.305	.658	.473	.234	.233	.310
General vision	.225	.266	.125	.164	.176	.116	.200	.231
Near vision*	.327	.404	.089	.123	.158	.294	.307	.299
Distance vision*	.392	.423	.145	.160	.183	.306	.344	.325
Driving	.448	.398	.186	.135	.019	.278	.285	.115
Peripheral vision	.195	.363	.279	.184	.253	.236	.238	.265
Color vision	.412	.313	.145	.207	.141	.297	.296	.204
Ocular pain	.276	.279	.306	.249	.269	.321	.266	.377
Vision-specific								
Role limitation	.340	.418	.173	.200	.200	.292	.376	.332
Dependency	.430	.447	.145	.179	.220	.383	.403	.390
Social function	.362	.405	.123	.118	.145	.313	.359	.294
Mental health	.346	.453	.192	.268	.235	.392	.382	.416
25-item composite	.448	.519	.222	.240	.264	.410	.441	.432

*Scores were recomputed after the substitution of items described in the text. Results of the recomputations are in parentheses.

The mean scores and the standard errors after adjustment for sex, age, and number of comorbid conditions are shown in Table 5. All scores were lower for those patients with age-related cataracts than for those in the reference group, with the exception of the 'Peripheral Vision', 'Color Vision', 'Ocular Pain', and 'Dependency' subscales. In addition, the subscales scores were significantly lower for those with ARMD than for those in the reference group, with the exception of the 'Peripheral Vision', 'Color Vision', and 'Ocular Pain' subscales. The item substitution described above resulted in slightly lower scores on the 'Near Vision' subscale and slightly higher scores on the 'Distance Vision' subscale. We tried the comparison of the explanation of variance caused by the influence of medical condition and visual acuity (Table 5). Two models associated with the NEI-VFQ score similarly.

**Table 5:** NEI VFQ-25 subscale scores and composite score, by condition* and the comparison of R2 between medical condition model and visual acuity model

					R^2 ^****	
					
Subscales	Cataract n = 96	Glaucoma n = 69	Age-related Macular Degeneration n = 78	Reference group n = 31	medical condition model	visual acuity model
General health	46.9 ± 2.0**	43.6 ± 2.3**	45.7 ± 2.4**	59.6 ± 4.4	.198	.209
General vision	56.0 ± 2.0**	62.7 ± 2.3**	41.2 ± 2.4**	74.0 ± 4.5	.273	.269
Near vision***	63.0 ± 2.6** (59.5 ± 5.4**)	69.5 ± 3.0 (67.9 ± 2.8)	38.6 ± 3.1** (31.9 ± 2.9**)	77.4 ± 5.8 (74.5 ± 5.4)	.341	.346
Distance vision***	59.1 ± 2.7** (65.1 ± 2.3**)	63.3 ± 3.1 (71.0 ± 2.7**)	40.0 ± 3.2** (47.4 ± 2.8**)	75.9 ± 6.0 (83.7 ± 5.1)	.268	.325
Driving	52.2 ± 5.5**	55.3 ± 6.0**	12.8 ± 5.2**	85.0 ± 10.9	.495	.424
Peripheral vision	57.3 ± 2.7	56.9 ± 3.1	64.1 ± 3.3	69.0 ± 6.1	.029	.126
Color vision	85.2 ± 2.1	89.6 ± 2.4	90.0 ± 2.6	88.1 ± 4.5	.081	.119
Ocular pain	80.5 ± 2.0	81.5 ± 2.4	83.2 ± 2.5	83.3 ± 4.6	.036	.100
Vision-specific						
Role limitation	71.3 ± 2.6**	73.5 ± 3.1	38.4 ± 3.2**	85.5 ± 5.9	.340	.252
Dependency	75.6 ± 2.8	83.9 ± 3.3	51.3 ± 3.5**	85.8 ± 6.3	.300	.306
Social function	73.5 ± 2.6**	80.0 ± 2.9	56.3 ± 3.1**	88.1 ± 5.6	.227	.252
Mental health	65.5 ± 2.5**	68.8 ± 2.9**	37.1 ± 3.1**	89.8 ± 5.7	.330	.300
25-item composite	66.0 ± 1.6**	69.8 ± 1.9**	51.0 ± 2.0**	80.1 ± 3.7	.331	.331

* Data are presented as mean ± SE adjusted by sex, age, and number of comorbid conditions.

** Significantly different from the reference group.

*** Scores were recomputed after the substitution of items described in the text. Results of the recomputations are in parentheses.

**** R2 showed the comparison of the explanation of variance caused by the influence of medical condition and visual acuity. The medical condition model have 'medical condition', 'sex', 'age', and 'number of comorbid condisions' as the explanatory variables and the visual acuity model have 'visual acuity', 'sex', 'age', and 'number of comorbid condisions'.

Visual acuity in the better eye (logMAR, the logarithm of the minimum angle of resolution) was strongly correlated with subscales that are influenced by the ability to use central vision: 'General vision', 'Near Vision', and 'Distance Vision' (Table 6). As would be expected, the logMAR was only weakly correlated with the subscales that are less dependent on the quality of central vision: 'General Health', 'Peripheral vision', 'Ocular Pain', and 'Color Vision'. In patients with glaucoma, visual field deficits were strongly correlated with scores on three subscales: 'Distance Vision', 'Driving', and 'Peripheral Vision' (Table 6). These correlations are similar to those observed between clinical measures and NEI VFQ scores in the NEI psychometric field test [9].

**Table 6:** Pearson correlations of NEI VFQ-25 subscale scores with visual acuity and visual field

Subscale	Visual acuity*		Visual field**	
	
	Better eye	Worse eye	Better eye	Worse eye
General health	0.06	0.06	0.03	-0.01
General vision	**0.55**	**0.50**	0.33	0.39
Near vision***	**0.60 (0.64)**	**0.56 (0.59)**	0.33 (0.34)	0.27 (0.30)
Distance vision***	**0.60 (0.59)**	**0.51 (0.52)**	**0.60 (0.54)**	**0.52 (0.41)**
Driving	**0.58**	**0.56**	**0.61**	**0.44**
Peripheral vision	0.06	0.06	**0.45**	**0.41**
Color vision	0.28	0.23	0.01	-0.06
Ocular pain	-0.02	-0.02	-0.15	-0.20
Vision-specific				
Role limitation	**0.51**	**0.46**	**0.36**	0.19
Dependency	**0.59**	**0.51**	**0.49**	**0.40**
Social function	**0.56**	**0.47**	**0.47**	0.28
Mental health	**0.55**	**0.49**	**0.49**	**0.41**
25-item composite	**0.61**	**0.54**	**0.49**	0.39

Bold characters indicate correlation coefficients of 0.4 or greater.

* Visual acuity (logMAR) while wearing usual correction, in all subjects in Sample 1

** Visual field testing with the Humphrey Field Analyzer 30-2 (only glaucoma, n = 69)

*** Scores were recomputed after the substitution of items described in the text. Results of the recomputations are in parentheses.

The results of factor analysis done with 10 subscales ('General Health' and 'Driving' were excluded) are shown in Table 7. Two factors were extracted. The 'Peripheral Vision', 'Ocular Pain', and 'Color Vision' subscales were included in the second factor. The correlation between the two factors was 0.47. The results of factor analysis done with 22 items (1 item on 'General Health' and 2 items on 'Driving' were excluded) had the similar to the structure of scale-level analysis.

**Table 7:** Results of factor analysis on 10 subscales of VFQ-25 ('General Health' and 'Driving' were excluded): factor loadings after promax rotation

Subscale	Factor 1	Factor 2
Near vision	**0.909**	-0.118
Mental health	**0.866**	0.052
Role limitation	**0.850**	-0.043
Dependency	**0.838**	0.053
Distance vision	**0.828**	0.067
General vision	**0.757**	-0.095
Social functioning	**0.724**	0.072
Peripheral vision	-0.033	**0.701**
Ocular pain	-0.094	**0.575**
Color vision	0.243	**0.421**

### Responsiveness

**Figure 1:**
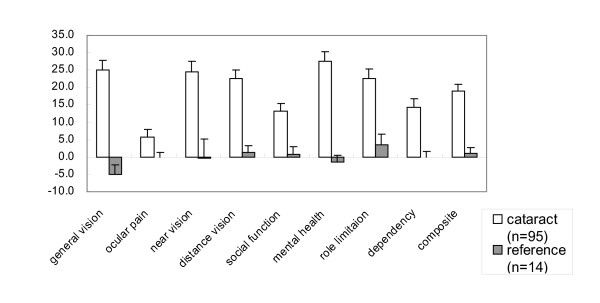
**Adjusted change in NEI VFQ-25 scores in the cataract-surgery group and the reference group. **Change score adjusted for sex and age.

The mean of visual acuity of patients with cataract were 20/200 before surgery, and after surgery it had improved to 20/50 in the better eye. In the reference group, scores were stable over two months. In the cataract surgery group, surgery was associated with significant increases in the composite score and in 8 subscale scores: 'General Vision', 'Near Vision', 'Distance Vision', 'Ocular Pain', 'Social Functioning', 'Mental Health', 'Role Limitation', and 'Dependency' (Figure 1). Guyatt's index of responsiveness for those subscale scores ranged from 1.91 to 7.35. Even the lower limit of that range would be considered to be extremely high [32]. The only exception was the 'General Health' subscale, which would not have been expected to be strongly influenced by cataract surgery.

On the basis of the result of factor analysis, we computed 3 different composite scores: composite 11 (all VFQ-25 subscales except 'General Health'), composite 10 (all VFQ-25 subscales except 'General Health' and 'Driving'), and composite 7 (only those 7 subscales of the VFQ-25 that loaded heavily on Factor 1, as indicated in Table 7). The responsiveness indexes of these three composite scores were 7.18, 8.03, and 8.86, respectively, all of which are acceptable from a psychometric perspective.

## Discussion

We developed a Japanese version of the NEI VFQ-25, and documented its psychometric characteristics in patients with various chronic eye conditions. Overall, we found that the Japanese version can provide data that are reliable, valid, and responsive to change in visual function.

In developing the Japanese version, a few changes to the content of the questionnaire were needed. For some of the items in the 'Near Vision' and 'Distance Vision' subscales, we found that the rates of missing data in the Japanese version were much higher than in the original English version. To minimize the rates of missing data and thereby to increase the measurement precision, we propose substituting items that are appropriate for patients in Japan. Specifically, instead of 'finding objects on crowded shelf', 'reading small print' can be used in the 'Near vision' subscale; and instead of 'going out to movies/plays', 'seeing television program' can be used in the 'Distance vision' subscale (both are from the pool of optional NEI VFQ items). Rates of missing data were much lower after those substitutions than before. The 'Near Vision' score was slightly higher and the 'Distance Vision' score was slightly lower, but their reliability and validity were virtually unchanged.

The 'Driving' subscale also had a high rate of missing data. We suggest that in Japan the 'Driving' subscale should be optional.

Composite scores can be useful summaries of visual function, particularly when the content of such a score is based on the results of factor analysis. In this study, factor analysis indicted that most of the subscales that are influenced by central vision correlated strongly with the first factor, while the 'Ocular Pain', 'Peripheral Vision', and 'Color Vision' subscales correlated strongly with the second factor. Therefore, if only one composite score is to be computed, that score should not include the 'Ocular Pain', 'Peripheral Vision', or 'Color Vision' subscales. Nonetheless, for studies of interventions involving small numbers of subjects we suggest using the 7-subscale composite score, given the caveat that it would not reflect problems with color vision, peripheral vision, or ocular pain. Furthermore, we suggest using the 10-subscale composite score when evaluating patients who have ocular pain or a disorder involving color vision.

Few reports [4,25] were available up until now on the responsiveness of the NEI VFQ-25. Almost no changes were observed in the VFQ-25 scores over 2 months in the reference group. In contrast, in the patients who underwent cataract surgery, many subscale scores increased by about 20 points. These increases occurred not only in the scores on subscales related directly to vision ('General Vision', 'Near Vision', and 'Distance Vision'), but also in the scores on subscales that are less vision-specific ('Mental Health', 'Dependency', 'Social Functioning', and 'Role Limitation'). Scores on the 'General Health', 'Color Vision', and 'Peripheral Vision' subscales did not change with cataract surgery. These results show that with the Japanese version of the NEI VFQ-25, one can easily detect clinically important changes such as those resulting from cataract surgery.

Interpretation of these results is limited in at least four ways. First, this study did not include patients with diabetic retinopathy, low vision, and a large number of other eye conditions. Thus, whether these findings are applicable to patients with diseases other than cataracts, glaucoma, and ARMD remains to be studied. Second, we used a convenience sample of persons with these conditions, and they may not represent the full clinical spectrum of each disease. Third, it is unclear whether the mode of administration (self-administered or interviewer administered) would have important effects on the results. However, we obtained the present data with trained interviewers, and we note that the findings are similar to those obtained in a field survey with the original English version, even though the questionnaire in that survey was self-administered. Fourth, the responsiveness results were obtained without the aforementioned substitutions in the 'Near Vision' and 'Distance Vision' subscales. Thus, the responsiveness of those two subscales should be examined again, after the recommended substitutions.

## Conclusion

In conclusion, psychometric testing indicates that data obtained with the Japanese version of the NEI VFQ-25 are sufficiently reliable, valid, and responsive for group-level comparisons. For reasons described in detail above, we suggest that a few items be substituted and that a few be removed from the composite score. Using this scale in vision-related clinical research in Japan should facilitate evaluations of clinical care and outcomes from the standpoint of the patient.

## Authors' contributions

Suzukamo Y assumed the coordination and design of this study, training of interviewers, data analysis and interpretation, and drafting this article. Oshika T, Yuzawa M, Tokuda Y, Tomidokoro A and Oki K contributed in the design of this study and acquisition of data. Mangione CM, Green J and Fukuhara S contributed in the concept and design of this study, interpretation of the data, and revising the article critically for important intellectual content.

## Supplementary Material

Additional File 1 Appendix: The list of items on the Japanese NEI-VFQ 25. Click here for file
