# Supplementary material for: Psychometric properties of the 25-item National Eye Institute Visual Function Questionnaire (NEI VFQ-25), Japanese version
Source: Health Qual Life Outcomes. 2005 Oct 26;3:65. doi: 10.1186/1477-7525-3-65 (PMC1283746; doi:10.1186/1477-7525-3-65)
Supplement: Additional File 1 — Appendix: The list of items on the Japanese NEI-VFQ 25. [file 1477-7525-3-65-S1.pdf]

Appendix: The list of items on the Japanese NEI-VFQ 25

Subscales	Number of items	Items
General health: GH	1	あなたの全身の健康状態はどうですか？
General vision: GV	1	現在、あなたの両眼での「ものの見えかた」は、どうですか？
Near vision: NV	3	ものが見えにくいために、新聞の記事を読むのは、どのくらい難しいですか？
		ものが見えにくいために、物を近くで見る作業（例えば料理や裁縫をしたり、家の中で修理をしたり工具を使ったり、など）をするのはどのくらい難しいですか？
		電話帳やくすりの説明書などの細かい文字を読むのは、どのくらい難しいですか？
Distance vision: DV	3	ものが見えにくいために、道路標識や商店の看板の文字を読むのは、どのくらい難しいですか？
		ものが見えにくいために、夜や薄暗いところで、階段をおりたり、歩道の段差をおりたりするのはどのくらい難しいですか？
		ものが見えにくいために、テレビ番組を見て楽しむのはどのくらい難しいですか？
Driving: DR	2	昼間、走り慣れた道を運転するのは、どのくらい難しいですか？
		夜間の運転はどのくらい難しいですか？
Peripheral vision: PV	1	ふだん道を歩くとき、ものが見えにくいために、まわりのものに気が付かないことがありますか？
Color vision: CV	1	ものが見えにくいために、その日に着る服を自分で選んだり、組み合わせたりするのはどのくらい難しいですか？
Ocular pain: OP	2	今まで、目や、目の周りに、痛みや不快感、例えば熱っぽさ、かゆみ、痛みなどは、どの程度ありましたか？
		目や、目のまわりの、痛みや不快感が原因で、やりたいことができないことがありますか？
Role limitations: RL	2	ものが見えにくいために、物事を思いどおりにやりとげられないことがありますか？
		ものが見えにくいために、仕事などのふだんの活動が長く続けられないことがありますか？
Dependency: DP	3	ものが見えにくいために、家にいることが多い。
		ものが見えにくいために、他の人が話すことにたよらなければならない。
		ものが見えにくいために、誰かの手助けを必要とすることが多い。
Social function: SF	2	ものが見えにくいために、あなたが何か言った時に相手がどう反応するかをみるのはどのくらい難しいですか？
		ものが見えにくいために、誰かの家を訪ねたり、何かの集まりやレストランに行ったりするのはどのくらい難しいですか？
Mental health: MH	4	自分の「ものの見えかた」について、不安を感じますか？
		ものが見えにくいために、欲求不満を感じる。
		ものが見えにくいために、したいことが思うようにできない。
		ものが見えにくいために、自分が気まずい思いをしたり、他の人を困らせたりするのではないかと心配である。

NEI－VFQ 25 Japanese version is available online at www.i-hope.jp (Japanese only)
